# Metabolomics of Hydrazine-Induced Hepatotoxicity in Rats for Discovering Potential Biomarkers

**DOI:** 10.1155/2018/8473161

**Published:** 2018-04-10

**Authors:** Zhuoling An, Chao Li, Yali Lv, Pengfei Li, Cheng Wu, Lihong Liu

**Affiliations:** ^1^Pharmacy Department of Beijing Chao-Yang Hospital Affiliated with Beijing Capital Medical University, Beijing, China; ^2^Pharmacy Department of the Second Artillery General Hospital of Chinese People's Liberation Army, Beijing, China

## Abstract

Metabolic pathway disturbances associated with drug-induced liver injury remain unsatisfactorily characterized. Diagnostic biomarkers for hepatotoxicity have been used to minimize drug-induced liver injury and to increase the clinical safety. A metabolomics strategy using rapid-resolution liquid chromatography/tandem mass spectrometry (RRLC-MS/MS) analyses and multivariate statistics was implemented to identify potential biomarkers for hydrazine-induced hepatotoxicity. The global serum and urine metabolomics of 30 hydrazine-treated rats at 24 or 48 h postdosing and 24 healthy rats were characterized by a metabolomics approach. Multivariate statistical data analyses and receiver operating characteristic (ROC) curves were performed to identify the most significantly altered metabolites. The 16 most significant potential biomarkers were identified to be closely related to hydrazine-induced liver injury. The combination of these biomarkers had an area under the curve (AUC) > 0.85, with 100% specificity and sensitivity, respectively. This high-quality classification group included amino acids and their derivatives, glutathione metabolites, vitamins, fatty acids, intermediates of pyrimidine metabolism, and lipids. Additionally, metabolomics pathway analyses confirmed that phenylalanine, tyrosine, and tryptophan biosynthesis as well as tyrosine metabolism had great interactions with hydrazine-induced liver injury in rats. These discriminating metabolites might be useful in understanding the pathogenesis mechanisms of liver injury and provide good prospects for drug-induced liver injury diagnosis clinically.

## 1. Introduction

Drug-induced liver injury is a life-threatening risk that is unpredictable and frequent during the clinical course of disease treatment [1–3]. The commonly used medicines as well as herbal products and vitamins may lead to liver injury [4–7]. The current methods for drug-induced liver injury diagnosis, such as blood biochemistry parameters including alanine aminotransferase (ALT), alkaline phosphatase (ALP), and aspartate aminotransferase (AST), are incapable of providing an accurate early diagnosis [8]. Hydrazine has been extensively applied as the classical animal model of liver injury and can also induce the alterations of endogenous metabolites including 2-aminoadipate, *β*-alanine, 2-oxoglutarate, and citrate [9–14]. However, to date, the reliability of biomarkers has not been verified and evaluated by multivariate data analysis methods or other methods of data analysis [15, 16]. Therefore, it is still necessary to identify and validate reliable biomarkers by using integrated detection methods and analysis methods for hydrazine-induced hepatotoxicity. Metabolomics is reported to be a powerful quantitative approach for determining global metabolite changes in response to disease or medical treatment and is particularly suitable for discovering biomarkers in a complex system [17–21]. Various techniques and methods have been applied for identification of endogenous metabolites for metabolomics analyses, including liquid chromatography-mass spectrometry (LC-MS) in addition to gas chromatography-mass spectrometry (GC-MS) and ^1^H nuclear magnetic resonance (^1^H-NMR) spectrometry [22–25]. The high sensitivity and potential for metabolite identification have made mass spectrometry (MS) the dominant approach for metabolomics studies [26, 27]. Previous research has shown that alterations of amino acids, glucose metabolism, lipid metabolism, and oxidative stress might be associated with hepatic fatty degeneration and glycogen accumulation, obtained mainly by using NMR- and GC-MS-based approaches [9–14]. These platforms or technologies have different strengths and weaknesses. Metabolomics studies currently lack a comprehensive metabolite identifying ability due to the lack of an electronic database. Multiple metabolomics platforms and technologies have allowed us to substantially enhance the level of metabolome coverage [28, 29]. Up to now, potential biomarkers for drug-induced injury lack methodology validation by other omics or metabolic pathways as well as large clinical samples, and the predictive ability of the analytical platform is not fully understood. Potential biomarkers capable of providing an accurate early diagnosis for drug-induced liver injury still require further mining. Thus, metabolomics based on LC-MS analyses may provide a comprehensive list of potential biomarkers for hydrazine-induced hepatotoxicity.

The present study utilized a nontargeted metabolomics approach by rapid-resolution liquid chromatography-mass spectrometry (RRLC-MS) combined with multivariate statistical analyses to investigate the metabolic profile changes between healthy and hydrazine-administered rats. Metabolic disturbances and changes in response to pathological conditions were further evaluated by multivariate statistics to identify potential diagnostic biomarkers. The reliabilities of the identified discriminated metabolites were further validated by receiver operating characteristic (ROC) analyses.

## 2. Experimental Procedures

### 2.1. Chemicals

Hydrazine hydrate was from Acros Organics (Morris Plains, NJ, USA). Acetonitrile and formic acid (HPLC-grade) were from Merck (Darmstadt, Germany). Metabolite standards with 99% purity (creatine, L-carnitine, tryptophan, tyrosine, kynurenic acid, adenosine, tanshinone IIA, peoniflorin, isosorbide 5-mononitrate, digitoxin, and tramadol) were from Sigma-Aldrich. All other standards used were of analytical or higher grade.

### 2.2. Animal Study Design and Sample Collection

Male Wistar rats (*n* = 60, 6-7 weeks old) were from Charles River China Inc. (Vital River Laboratories, China). The experiments were carried out under the approval by the Animal Ethics Committee of Beijing Chao-Yang Hospital affiliated with Beijing Capital Medical University. Rats were randomly divided into four groups: two control groups (12 rats/group) and two hydrazine-treated groups (18 rats/group). One control group and one hydrazine-treated group were allocated for sampling at 24 h postdosing, while the remaining two groups were for sampling at 48 h postdosing. The hydrazine-treated groups were orally administrated with a single dose of hydrazine (150 mg/kg), at which hydrazine could induce an obvious histopathological effect and hepatocellular lipid accumulation [10–14].

Urine samples from metabolic cages were collected for 8 h from 16 to 24 h and 40 to 48 h postdosing and then centrifuged at 5000 ×g for 5 min at 4°C; the supernatant was used for creatinine concentration measurement [14].

The orbital blood was taken from rats at 24 and 48 h postdosing. Serum samples collected by centrifugation at 5000 ×g, and 4°C for 5 min was used for determining ALT, ALP, and AST concentrations. Blood biochemistry analyses were measured by a clinical biochemistry analyzer (AU400, Olympus, Japan) at De Yi Biotechnology Co. Ltd.

After blood collection, the rat livers were collected and fixed in 4% formaldehyde. The fixed rat liver samples were trimmed, embedded in paraffin wax, sectioned, and then stained with hematoxylin and eosin (H&E) for histopathological examination.

### 2.3. Sample Preparation

An aliquot of 50 *μ*L of urine supernatant was further diluted to 200 *μ*L with water and then filtered through a syringe filter (0.2 *μ*m, 96-well Captiva sample prep solutions, Agilent Technologies, USA) before LC-MS analysis. A 100 *μ*L aliquot of serum was added to 300 *μ*L of cold acetonitrile, and the mixture was vortexed for 4 min and centrifuged at 12,000 ×g for 10 min. A total of 200 *μ*L of the supernatant was centrifuged (SAVANT SPD121 P SpeedVac, Thermo Scientific, USA) for 60 min or 90 min at 4°C. Dry residues were reconstituted in 200 *μ*L of water containing 2% acetonitrile and were vortexed for 5 min. The supernatant was removed and filtered through a syringe filter for RRLC-MS analysis.

### 2.4. RRLC-MS Analysis

Chromatographic separation was performed on an ACQUITY UPLC HSS T_3_ column (1.8 *μ*m, 10 cm × 2.1 mm, Waters, Ireland) with an Agilent 1200 RRLC system (Agilent Technologies, Aldbronn, Germany). The column temperature was kept at 50°C, and the injection volume was 5 *μ*L. The analysis gradient conditions for both serum and urine samples were the same, as follows: 0–5 min, linear gradient of 2–10% B; 5–17 min, linear gradient of 10–40% B; 17–20 min, 40–100% B; and 20–30 min, 100% B. The program was followed by a return to the starting conditions, which were maintained for 8 min to equilibrate the column. Mobile phase A was 0.1% formic acid in water, and mobile phase B was acetonitrile. The flow rate was 250 *μ*L/min.

MS spectra were acquired on a Q-TOF LC-MS system (AB/MDS Sciex, Foster City, CA) with an electrospray ion (ESI) source in positive-ion mode. The ion source voltage was 5.5 kV, and the declustering potential was 50 V. The collision energy (CE) was set to 30/10 eV, and the vaporizer temperature was 450°C. The MS was operated with gas conditions of 30 psi for the curtain gas, 5 psi for the collision gas, 70 psi for the nebulizer gas (GS1), and 60 psi for the drying gas (GS2). Nitrogen gas was used as both the nebulizing and drying gas. The mass-to-charge (*m*/*z*) scan range was from 65 to 1000. Data processing and acquisition were conducted on Analyst QS 2.0 software (QSTAR Elite, AB/MDS Sciex).

MS/MS spectra were obtained with information-dependent acquisition mode. MS signals were first collected by one time-of-flight (TOF) survey scan and followed by two product-ion scans for parent ions with the highest intensity. For each survey scan, three MS/MS experiments were triggered. Compounds with identical *m*/*z* ratios were excluded automatically. Metabolites of interest were added to the MS/MS table list. MS/MS data were auto-normalized by background ions (phthalates: *m*/*z* 391.2843 and 149.0233).

### 2.5. Assessment for Data Quality

To obtain high-quality and reliable data to reflect the endogenous metabolite changes, analytical and technical errors should be decreased to minimize any influence on multivariate data analysis. In the current study, a test standard mixture, including adenosine, tanshinone IIA, peoniflorin, isosorbide 5-mononitrate, digitoxin, and tramadol, was added into the sample batch to monitor the system stability. Quality control (QC) samples prepared by pooling equal volumes of urine or serum from each healthy and liver-injured rat were treated as the real samples to monitor system reproducibility and stability [27].

### 2.6. Data Processing

RRLC-MS raw data were converted to the *m*/*z* format with the threshold set at 1% using wiff to *m*/*z* data translator software (version 1.0.0.4, AB/MDS Sciex). Using open-source software MZmine 2 beta (version 2.9.1), peak finding, alignment, filtering, and scaling were carried out. All parameters were optimized in a stepwise manner until aligned peaks agreed with those from manual inspection [30, 31]. Detailed preprocessing parameters are listed in S1 Table [22].

The preprocessing data from urine samples were normalized by the creatinine level. The obtained data were then subjected to SIMCA-P 13.0 software (Umetrics AB, Umeå, Sweden) for multivariate data analysis. In order to reduce model noise and artifacts, centered- and pareto-scaling were used prior to multivariate statistical analysis. To test the model validity against overfitting and to validate the biomarkers statistically, *Q* ^2^, the cross-validation parameter, was calculated using partial least squares discriminant analysis (PLS-DA) by a random permutation test of 100 permutations. Discriminating variables were chosen based on jack-knife confidence intervals, S-plot, variable importance on projection (VIP) values, and raw data plots using the orthogonal partial least squares discriminant analysis (OPLS-DA) model. The OPLS-DA model quality was evaluated by the relevant *R* ^2^ and *Q* ^2^ values as well as the intercepts of *R* ^2^ and *Q* ^2^. The OPLS-DA models were considered to be valid only if 0 < *R* ^2^(*Y*)–*Q* ^2^(*Y*) < 0.3, *Q* ^2^(*Y*) > 0.5, intercepts of *R* ^2^ < 0.4, and intercepts of *Q* ^2^ < 0.05 [32]. The independent *t*-test (2007 version of Microsoft Office Excel) was used to check whether potential biomarkers derived from OPLS-DA modeling were statistically significant (*p* < 0.05).

Metabolites were identified via searching free databases including HMDB, PubChem, METLIN, and KEGG, using accurate molecular weights [33]. High-resolution mass spectra (HR-MS/MS) were applied for further identification. Based on the retention time comparison, authentic standards were also used. Each identified biomarker was evaluated for its discriminatory power and predictive ability using the area under the receiver operating characteristic curve [34]. Metabolic pathway analysis for potential biomarkers was carried out by powerful pathway enrichment analysis and pathway topology analysis to identify the most relevant pathways through MetaboAnalyst 3.0 (http://www.metaboanalyst.ca). The most relevant pathways involved were confirmed only if the pathway impact was greater than 0.1 and the node size and color were graphically larger and darker.

## 3. Results and Discussion

### 3.1. Toxicological Examination

Hydrazine-induced hepatotoxicity in rats was confirmed by evaluation of blood biochemistry and liver tissue histology. Alternations of the blood biochemical parameters between healthy and hydrazine-treated rats are listed in Table 1. AST decreased significantly in the hydrazine-treated rats at both 24 and 48 h postdosing, perhaps owing to hydrazine or its metabolites causing sequestration of the aminotransferase cofactor pyridoxal 5-phosphate, resulting in inhibitory effects on aminotransferase activities [9–14, 35]. However, ALT and ALP did not significantly differ between hydrazine-treated groups at 24 and 48 h postdosing and their controls, respectively, indicating hepatic injuries might be accompanied with little changes of ALT and ALP for self-regulation and recovery. The results also suggested that the activities of hepatotoxicity enzymes might not be sensitive enough to predict hydrazine-induced liver injury.

**Figure 1:**
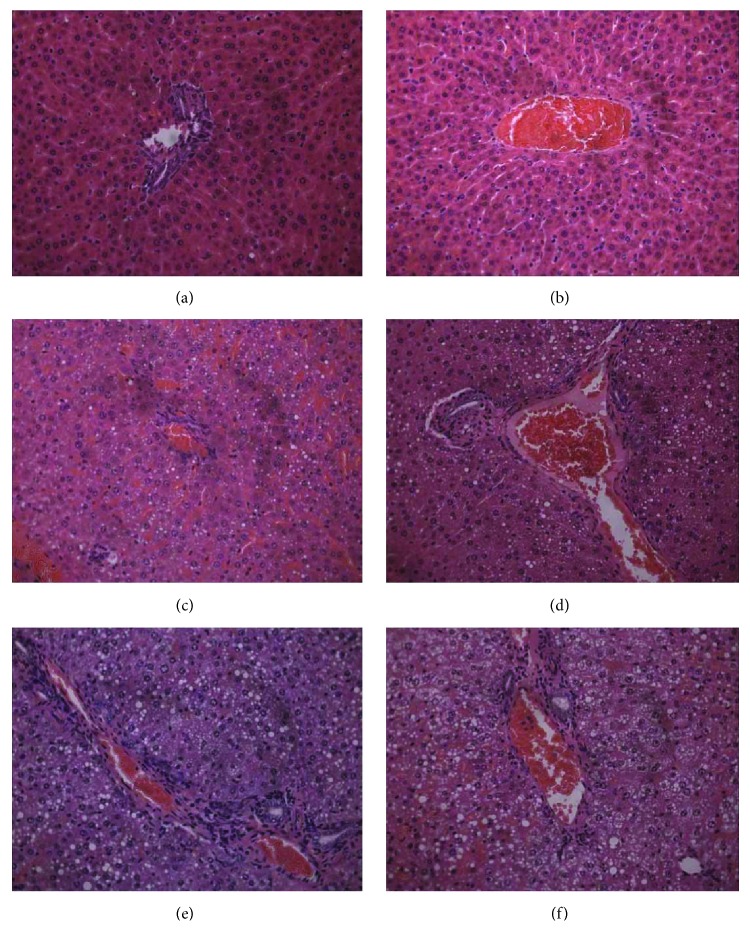
Liver histopathology at 24 h and 48 h postdosing for control rats and liver-injured rats, which were induced by hydrazine. No abnormalities were detected in the controls (0 mg/kg) at the 24 h and 48 h time points (a and b). Fatty degeneration and single-cell necrosis appeared obviously in the midzonal areas of the hydrazine-treated groups (150 mg/kg) at 24 h (c and d) and 48 h (e and f) postdosing. Histological sections were stained with H&E (×400).

The histopathological photographs are shown in Figure 1. Figures 1(a) and 1(b) show normal hepatocytes surrounding the central vein in control rats, and Figures 1(c)–1(f) show the cytoplasmic vacuoles induced by fatty degenerations in the midzonal area of hepatocytes and single-cell necrosis of hepatocytes in hydrazine-treated rats. Additionally, isolated extracellular and eosinophilic bodies were distributed in some necrotic cells of hydrazine-treated rats. These findings indicate that hydrazine-treated rats at 24 or 48 h postdosing had suffered from serious liver injury.

### 3.2. Assessment of Data Quality

To obtain high-quality and reliable data to reflect the endogenous metabolite changes, analytical and technical errors should be decreased to minimize any influence on multivariate data analysis. In the current study, a test standard mixture including adenosine, tanshinone IIA, peoniflorin, isosorbide 5-mononitrate, digitoxin, and tramadol was added into the sample batch to monitor the system stability. As demonstrated in S1 Fig, the maximum deviation in retention time for all standards in the test standard mixture was 0.06 min, the average variation of retention time was <0.82% (RSD), and the average variation of extracted ion areas was <14.30% (RSD) (S2 Table). Multivariate analysis was performed after creatinine normalization and unit variance scaling. Using principal component analysis (PCA), the peak area deviation of serum or urine QC plots was less than 2-fold of the SD (S2 Fig). These results indicate that differences between test samples were more likely to reflect varied metabolic profiles than technical errors or analytical variation.

### 3.3. Multivariate Statistical Analysis of Control and Liver-Injured Rats

Using MZmine software, the RRLC-MS data of urine and serum samples were analyzed by peak detection and integration, leading to the identification of *m*/*z* 647 and *m*/*z* 604 peaks for urine and serum samples between retention times of 1 to 25 min, respectively.

**Figure 2:**
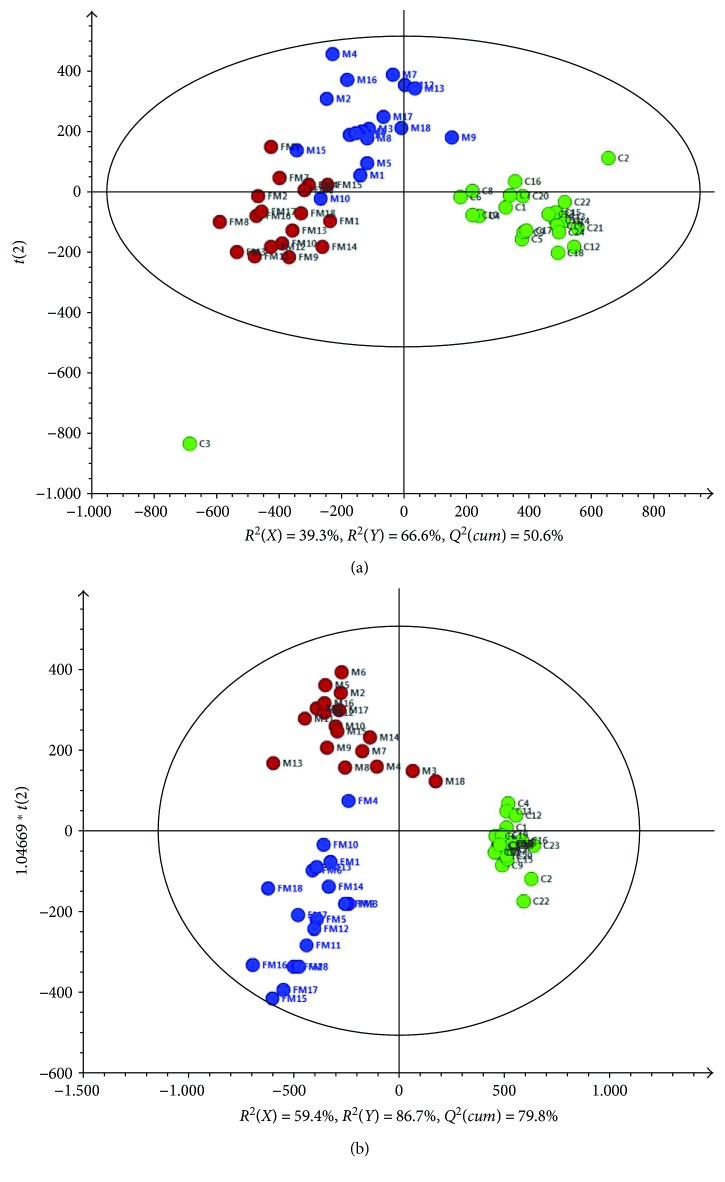
OPLS-DA score plots of (a) serum and (b) urine samples derived from the RRLC−(+) ESIMS data. (The symbols are as follows: green circle = C healthy rats; blue circle = M liver-injured rats induced by hydrazine at 24 h postdosing; and brown circle = FM liver-injured rats induced by hydrazine at 48 h postdosing.)

**Figure 3:**
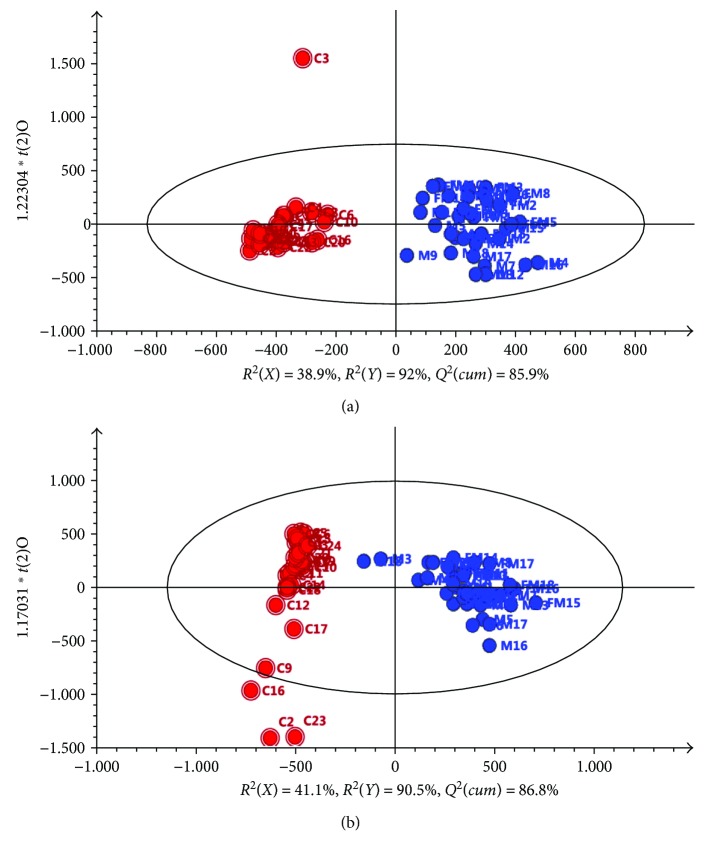
OPLS-DA score plots derived from the RRLC−(+) ESIMS data from (a) serum and (b) urine samples. (The symbols are as follows: red circle = C healthy rats and blue circle = M and FM liver-injured rats induced by hydrazine at 24 h and 48 h postdosing.)

First, PCA was applied to integrate and coanalyze all observations from serum and urine samples to investigate metabolomics changes. The control groups and hydrazine-treated groups at both 24 h and 48 h postdosing were not well distinguished in terms of PCA score plots, and the 48 h postdosing group did not show a significant difference compared with the 24 h postdosing group, especially for the urine samples. OPLS-DA was applied to the control groups and hydrazine-treated groups at both 24 h and 48 h postdosing. The separation of the three groups was good (Figure 2). Furthermore, OPLS-DA was applied as a stoichiometric analysis method to explore differences between healthy and liver-injured rats. Score plots for the OPLS-DA models showed a clear separation between healthy and liver-injured rats (Figure 3). For serum data analyses, one predictive component (*t*_p_) and two orthogonal (*t*_o_) components (1 + 2) with cross-validated predictive abilities *Q* ^2^(cum) of 85.9% were derived. Additionally, 38.9% of the variance in *X*[*R* ^2^(*X*)] was applied to account for 92% of the *Y*[*R* ^2^(*Y*)] variance (Figure 3(a)). For urine data analyses, *R* ^2^(*X*) = 41.1%, *R* ^2^(*Y*) = 90.5%, and *Q* ^2^(cum) = 86.8% across one predictive component (*t*_p_) and two orthogonal (*t*_o_) components are shown in Figure 3(b).

To avoid overfitting, a seven-round validation across three components by excluding 1/7th of the samples in each round was applied. Validation by 100 random permutation tests resulted in intercepts of *R* ^2^ = 0.267 and *Q* ^2^ = −0.235 for the serum data (S3 Fig A and B). Similarly, the validated intercepts of *R^2^* = 0.213 and *Q^2^* = −0.242 were calculated for the urine data (S3 Fig C and D). These results demonstrated that the OPLS-DA models based on the RRLC-ESI-MS data showed good predictability for both the serum and urine samples.

### 3.4. Screening of Potential Diagnostic Biomarker Candidates

S-plot was applied to screen the discriminative variables. Metabolites with a high correlation were selected preferentially [36]. Variables with a VIP > 1.0 were marked as variables of interest [37]. Raw data plots and jack knife-based confidence interval analyses were subsequently employed to remove variables with low reliability. In parallel, the independent *t*-test was applied to evaluate the significance of the concentration difference for identified variables (*p* < 0.001). Redundant variables obtained from the same metabolite were excluded by partial correlation coefficients. Consequently, 27 and 53 metabolites detected in serum and urine, respectively, were deemed to be biomarker candidates reflecting metabolic differences. To further identify serum- or urine-specific biomarker(s), the first screened potential biomarkers were evaluated by a self-compiled Microsoft Visual Basic program [22]. The results showed that only five discriminatory metabolites were identified in both serum and urine. In total, 22 and 48 metabolites were considered as unique biomarkers for serum and urine, respectively (S3 Table).

### 3.5. Identification of Potential Biomarkers by MS/MS Fragmentation Patterns

**Figure 4:**
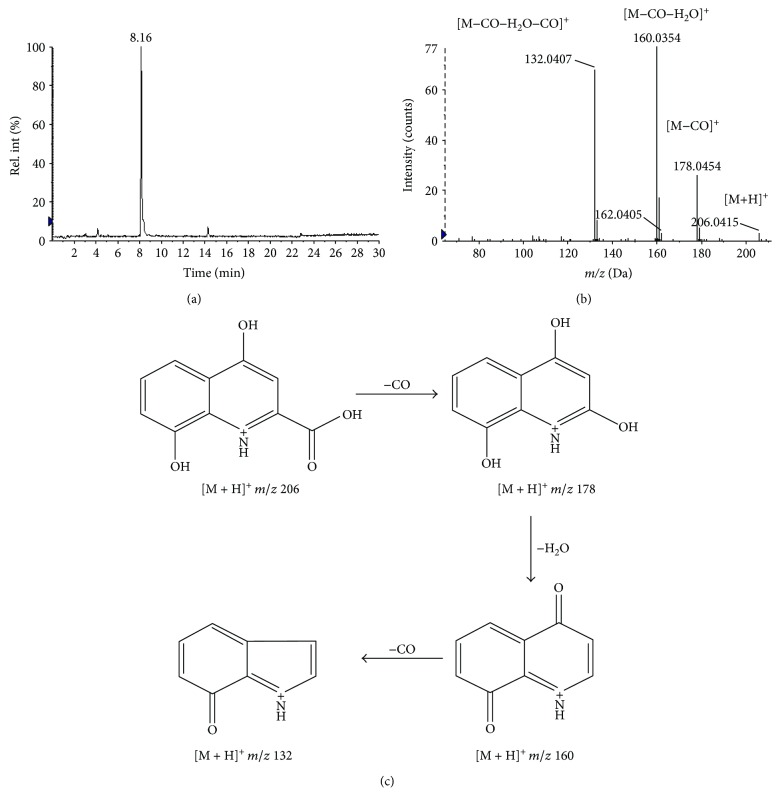
(a) Extracted ion chromatogram (XIC) of *m*/*z* 206.0415 in positive-ion mode of LC-MS analysis for urine samples. (b and c) The identification of the metabolite xanthurenic acid by means of Q-TOF MS/MS in positive-ion mode; the positive product ion spectrum of *m*/*z* 206.0415 at 8.16 min (b) and its postulated main fragmentation pathway (c).

A molecular formula could be determined according to the accurate mass weights, mass defect considerations, assignments, MS/MS fragmentation patterns, and relative intensities of the isotope peaks obtained from the HR-MS/MS [22, 31, 33]. Here, the identification processes were briefly demonstrated by taking *m*/*z* 206.0415 as an example (Figure 4). Such steps enabled the characterization of 19 potential biomarkers in the serum and urine. Their HR-MS/MS fragments and relative abundances are listed in Table 2. Five potential screened biomarkers (creatine, l-carnitine, tryptophan, tyrosine, and kynurenic acid) were further confirmed by the retention time comparison with the standard.

### 3.6. Characterization of Potential Diagnostic Biomarkers

**Figure 5:**
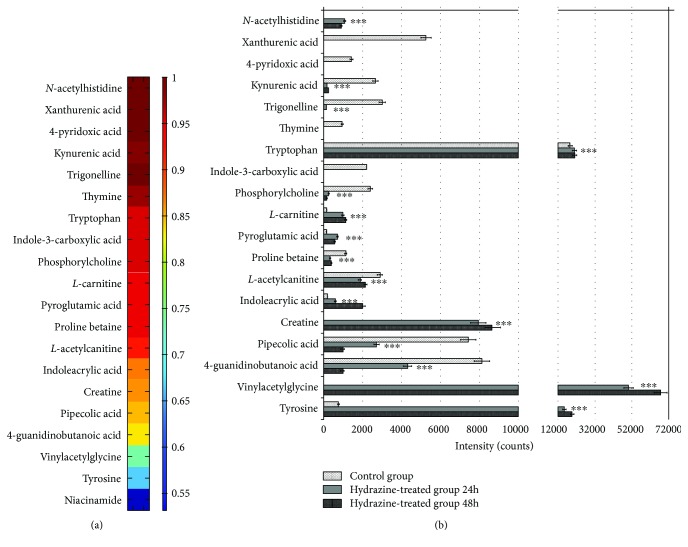
(a) Visualization of the discriminatory powers of individual potential biomarkers (AUC values > 0.8). Heat map showing the discriminatory capacity of each metabolite estimated by the AUC. Colors correspond to AUC values; red and blue represent high and low values, respectively. (b) Concentration changes of potential biomarkers in the control and liver-injured groups (^∗∗∗^ *p* < 0.001).

ROC analyses were performed to reconfirm these putative biomarkers for their discriminatory power. Nineteen identified potential biomarkers (8 for serum, 12 for urine, and 1 for both serum and urine) were classified into two categories, 8 were upregulated, and 11 were downregulated in liver-injured rats (Table 2). The seven upregulated metabolites had AUC values of 0.667 to 1 (S4 Fig A). Only tyrosine showed a relatively low diagnostic significance (AUC < 0.7) [31, 34]. The eleven downregulated metabolites provided AUC values of 0.838 to 1 (S4 Fig B). Heat maps were also obtained to evaluate the discriminative power of potential biomarkers, and their AUC values are ranked in Figure 5(a). The peak area changes between the control and hydrazine-treated groups are summarized in Figure 5(b). To achieve more discriminative power, a biomarker group was generated including 6 upregulated (creatine, tryptophan, *N*-acetylhistidine, l-carnitine, pyroglutamic acid, and indoleacrylic acid) and 10 downregulated (prolinebetaine, l-acetylcarnitine, pipecolic acid, xanthurenic acid, trigonelline, kynurenic acid, indole-3-carboxylic acid, phosphorylcholine, 4-pyridoxic acid, and thymine) metabolites with AUC > 0.85. These biomarkers were further analyzed by binary logistic regression, followed by ROC curve analysis, which provided an AUC of 1. In addition, both specificity and sensitivity calculated at best cut-off points could reach 100% (S5 Fig). These findings demonstrate that this group of combined biomarkers showed a more preferable discrimination capability between healthy and liver-injured rats.

### 3.7. Biological Significance of the Identified Potential Biomarkers

Tyrosine and tryptophan have been shown to be associated with thioacetamide-induced liver fibrosis [38]. In the tryptophan metabolism pathway, lower levels of kynurenic acid and xanthurenic acid have been confirmed to indicate disturbances in liver function and energy-related metabolism [39, 40]. Furthermore, downregulation of indoleacrylic acid and indole-3-carboxylic acid might result from a tryptophan metabolism disorder involved in liver diseases [41].

Elevated creatine kinase and transaminase levels have been found in statin-induced liver damage [42]. In the current study, a dramatic decrease of urinary pipecolic acid was observed in hydrazine-induced liver injury. In addition, the upregulation of plasma pipecolic acid has been reported in chronic liver disease patients almost paralleling the severity of liver damage [43]. Pyroglutamic acid, an intermediate of the endogenous tripeptide glutathione biosynthesis pathway, is well documented as a hepatotoxicity biomarker following acetaminophen exposure [10, 44].

Trigonelline is an effective lipid-lowering agent that occasionally causes hepatic failure [45]. Previous studies have demonstrated that trigonelline is decreased by acute and chronic acetaminophen administration [46]. 4-Pyridoxic acid, the catabolic product of vitamin B6, regulates transsulfuration reactions for glutathione production and aberrant vitamin B-dependent hepatic methionine metabolism [47]. Moreover, 4-pyridoxic acid, indoleacrylic acid, and tryptophan have been shown to have a great influence on oxidative stress as well as on liver and renal dysfunction induced by pesticides [48].

Compared with the healthy controls, the liver-injured rats demonstrated significantly higher serum levels of l-carnitine, while the level of acetylcarnitine decreased. Carnitine is reported to be essential for fatty acid metabolism and transport of activated long-chain fatty acids to *β*-oxidation sites in the mitochondria [49]. In rats, l-acetylcarnitine has been shown to increase the endogenous antioxidant defense mechanism and thus protect the animals from radiation-induced liver toxicity [50]. Therefore, elevated carnitine and decreased acylcarnitine levels might be important indicators for oxidation disturbances of long-chain fatty acids that are closely related to liver injury. Furthermore, increased levels of l-dihydroorotic acid and decreased levels of thymine have been demonstrated to result from a pyrimidine metabolism disorder in the development of liver diseases [51].

**Figure 6:**
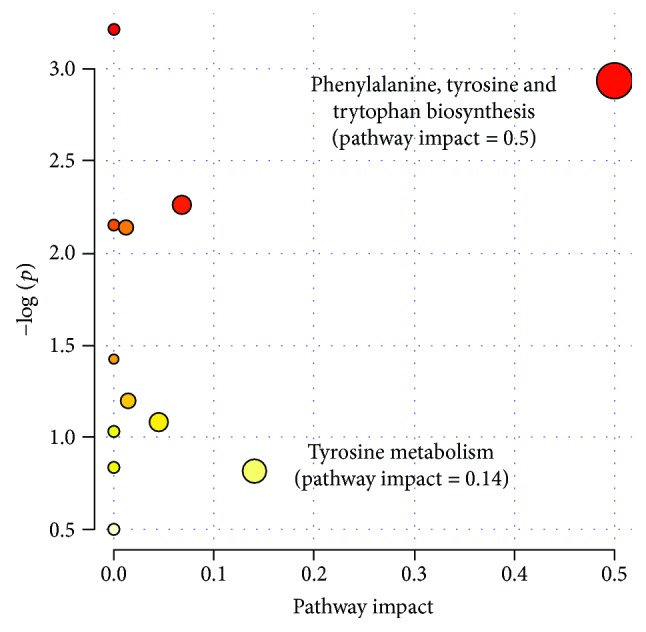
Metabolic pathway analysis for potential biomarkers related to liver-injured rats induced by hydrazine. The most relevant pathways are represented by large and dark nodes (pathway impact > 0.1).

Elevated phosphorylcholine concentrations were observed in human hepatic tumor patients due to reconstitution of phospholipids in the injured membrane, and the opposite change was found in liver-injured rats [52]. These potential biomarkers indicate that perturbations of amino acids, glutathione metabolism, vitamins, fatty acids, pyrimidine, and lipid metabolism might be important in liver dysfunction. Additionally, metabolomics pathway analyses confirmed that phenylalanine, tyrosine, and tryptophan biosynthesis as well as tyrosine metabolism had great interactions in hydrazine-induced liver injury in rats (Figure 6).

## 4. Conclusions

We investigated the application of an RRLC-MS/MS-based metabolomics method for hepatotoxicity evaluation in rats. To achieve a better discriminatory capability, a biomarker group was built by assembling biomarkers with high diagnostic value based on ROC analysis and a logistic regression model. The sensitivity and specificity of prediction were significantly improved by a group of biomarkers consisting of seven upregulated and ten downregulated metabolites. These discriminating metabolites might be useful in understanding the pathogenesis mechanisms of liver injury and provide good prospects for drug-induced liver injury diagnosis clinically.

This study confirmed the feasibility of using an LC-MS-based urine metabolomics platform to characterize hydrazine-induced hepatotoxicity in rats. However, the specificity of these potential biomarkers must be further evaluated. Further studies will be applied to validate these biomarkers in larger cohorts of different patients.

## Figures and Tables

**Table 1:** Alterations of blood biochemistry parameters in hydrazine-treated rats.

Time	Dose	ALT (U/L)	ALP (U/L)	AST (U/L)
24 h	0 mg/kg	48.4 ± 14.8	199.5 ± 21.4	150.7 ± 34.9
	150 mg/kg	39.8 ± 10.2	180.1 ± 24.1^∗^	109.8 ± 55.5^∗^

48 h	0 mg/kg	47.3 ± 19.4	193.1 ± 41.0	131.2 ± 18.0
	150 mg/kg	31.3 ± 15.5^†^	201.1 ± 27.5	86.6 ± 45.6^††^

ALT: alanine aminotransferase; ALP: alkaline phosphatase; AST: aspartate aminotransferase. ^∗^Significantly different from the 0 mg/kg group (24 h) (*p* < 0.05). †Significantly different from the 0 mg/kg group (48 h) (*p* < 0.05). ††Significantly different from the 0 mg/kg group (48 h) (*p* < 0.01).

**Table 2:** Identified potential biomarkers related to perturbations of hydrazine-induced liver injury.

Biological sample	RT (min)	*m*/*z*	Elemental composition	Metabolite identification	MS/MS fragments*^c^*	VIP*^d^* (trend)	*p* value*^e^*
Serum	2.19	130.0512	C_5_H_8_NO_3_^+^	Pyroglutamic acid*^b^*	84.0449	1.03 ↑	2.33*E* − 09
Serum	1.22	132.0770	C_4_H_10_N_3_O_2_^+^	Creatine*^a,b^*	90.0555, 87.0791, 72.0565	4.25 ↑	9.83*E* − 11
Serum	1.26	144.1029	C_7_H_14_NO_2_^+^	Prolinebetaine*^b^*	102.0556, 84.0812	1.36 ↓	3.44*E* − 14
Serum	1.16	162.1129	C_7_H_16_NO_3_^+^	l-Carnitine*^a,b^*	103.0377, 85.0277	1.45 ↑	3.15*E* − 13
Serum	1.14	198.0861	C_8_H_12_N_3_O_3_^+^	*N*-Acetylhistidine*^b^*	153.0890, 138.0548	1.62 ↑	1.86*E* − 20
Serum	1.80	204.1243	C_9_H_18_NO_4_^+^	l-Acetylcarnitine*^b^*	144.1032, 85.0870	1.25 ↓	1.28*E* − 06
Serum	5.20	205.0990	C_11_H_13_N_2_O_2_^+^	Tryptophan*^a,b^*	188.0704, 146.0612, 118.0673	2.30 ↑	1.21*E* − 09
Serum/urine	2.62	182.0803	C_9_H_12_NO_3_^+^	Tyrosine*^a,b^*	165.0548, 136.0760, 123.0449, 119.0490	5.38/2.28 ↑	1.77*E* − 06/9.94*E* − 04
Urine	1.51	127.0490	C_5_H_7_N_2_O_2_^+^	Thymine*^b^*	84.0444	1.11 ↓	5.05*E* − 11
Urine	1.69	130.0857	C_6_H_12_NO_2_^+^	Pipecolic acid*^b^*	84.0808	2.28 ↓	9.68*E* − 06
Urine	1.21	138.0534	C_7_H_8_NO_2_^+^	Trigonelline*^b^*	94.0650, 78.0332, 67.0407	1.96 ↓	3.43*E* − 12
Urine	4.02	144.0660	C_6_H_11_NO_3_^+^	Vinylacetylglycine*^b^*	99.0680, 98.0568, 86.0600	7.35 ↑	1.55*E* − 05
Urine	1.70	146.0913	C_5_H_12_N_3_O_2_^+^	4-Guanidinobutanoic acid*^b^*	111.0566, 87.0440, 86.0584, 69.0326	2.42 ↓	1.14*E* − 06
Urine	11.1	162.0532	C_9_H_8_NO_2_^+^	Indole-3-carboxylic acid*^b^*	144.0414, 116.0463, 89.0382	1.62 ↓	2.00*E* − 09
Urine	4.09	184.0590	C_8_H_10_NO_4_^+^	4-Pyridoxic acid*^b^*	166.0469, 148.0400	1.41 ↓	4.19*E* − 14
Urine	6.91	185.1245	C_5_H_16_NO_4_P^+^	Phosphorylcholine*^b^*	126.0902	1.55 ↓	7.13*E* − 08
Urine	7.97	188.0692	C_11_H_10_NO_2_^+^	Indoleacrylic acid*^b^*	142.0651	1.05 ↑	1.71*E* − 06
Urine	8.82	190.0482	C_10_H_8_NO_3_^+^	Kynurenic acid*^a,b^*	162.0527, 144.0431, 116.0464, 89.0357	1.86 ↓	2.86*E* − 15
Urine	8.16	206.0430	C_10_H_8_NO_4_^+^	Xanthurenic acid*^b^*	178.0462, 160.0367, 132.0415	2.22 ↓	9.00*E* − 06

*^a^*Metabolites confirmed using standard compounds. *^b^*Metabolites confirmed by literature or database searches and MS fragmentation. *^c^*MS/MS fragments were obtained with a CE of 30/10 eV, respectively. *^d^*VIP is variable importance in the projection obtained from OPLS-DA with a threshold of 1.0. *^e^p* value of the independent *t*-test between the control and model group.

## Supplementary Materials

Supplementary Materials S1 Table: the parameters of detailed preprocessing data applied in MZmine. S2 Table: method precisions (RSD) (*n* = 6) of LC-MS analysis for serum and urine samples. S3 Table: summary of potential biomarkers found between serum and urine samples of healthy rats and liver-injured rats by RRLC-ESIMS analysis in positive-ion mode. S1 Fig: overlaid extracted ion chromatograms of standard compounds in various test mixtures using RRLC-MS analysis during the whole sample batch (*n* = 6). Six standard compounds, (a) adenosine, (b) tanshinone IIA, (c) tramadol, (d) isosorbide mononitrate, (e) digitoxin, and (f) telmisartan, were selected to prepare various test mixtures for the (A) serum and (B) urine samples. They all provide an overall idea of the system stability during the run. S2 Fig: quality control (QC) plots of six randomly repeated runs of RRLC–(+) ESIMS generated by principal component analysis for serum (A) and urine (B). Peak area deviation could be evaluated by the distribution of the runs. *x*-axis: run order; *y*-axis: standard deviation. (A1) and (B1) are QC plot of principal component analysis; (A2) and (B2) are QC plot for the first component. (A3) and (B3) are QC plot for the second component. S3 Fig: validation plots of the PLS-DA models obtained by using 100 permutation tests and T-predicted scatter plots of the OPLS-DA model for serum (A-B) and urine (C-D) samples. (The symbols are as follows: red circle = C healthy rats; brown circle = M liver-injured rats induced by hydrazine at 24 h postdosing; and blue circle = FM liver-injured rats induced by hydrazine at 48 h postdosing.) S4 Fig: visualization of the discriminatory powers of potential biomarkers. (A) The eight upregulated metabolites provided AUC values of 0.667 to 1. (B) The 11 downregulated metabolites provided AUC values of 0.838 to 1. S5 Fig: ROC curve of the combined potential biomarkers (AUC > 0.85) that provided an AUC of 1 and sensitivities and specificities that reached 100%, respectively.
