# Supplementary material for: Metabolomics of Hydrazine-Induced Hepatotoxicity in Rats for Discovering Potential Biomarkers
Source: Dis Markers. 2018 Apr 10;2018:8473161. doi: 10.1155/2018/8473161 (PMC5914126; doi:10.1155/2018/8473161)

**Supporting Information**

Metabolomics of hydrazine-induced hepatotoxicity in rats for discovering potential biomarkers

Zhuoling An^1^, Chao Li^2^, Yali Lv^1^, Pengfei Li^1^, Cheng Wu^2^, Lihong Liu^1*^

1 Pharmacy Department of Beijing Chao-Yang Hospital affiliated with Beijing Capital Medical University, Beijing, PR China,

2 Pharmacy Department of the Second Artillery General Hospital of Chinese People’s Liberation Army, Beijing, PR China

Correspondence

Lihong Liu, Professor of Medicine

8 Gongren Tiyuchang Nanlu, Chaoyang District, Beijing.

Tel: (+86) 01085231788.

Fax: (+86) 01085231788.

e-mail: hongllh@126.com.

**S1 Table. The parameters of detailed data pre-processing applied in MZmine**

**S2 Table. Method precisions (RSD) (*n*=6) of LC-MS analysis for serum and urine**

**S3 Table. Summary of potential biomarkers found between healthy rats and liver injury group in serum and urine by RRLC-ESIMS analysis in positive modes**

| **S1 Table. The parameters of detailed pre-processing data applied in MZmine.** | | | |
| --- | --- | --- | --- |
| Settings | | serum (+)ESI-MS | Urine (+)ESI-MS |
| Centroid mass detector | Noise level (counts) | 50 | 50 |
| High data point chromatogram construction | Minimum time span (s) | 2 | 2 |
|  | Minimum height(counts) | 20 | 20 |
|  | *m/z* tolerance size | 0.1 | 0.1 |
| Baseline peak recognizer | Minimum acceptable height (counts) | 50 | 50 |
|  | Minimum peak duration (s) | 2 | 2 |
|  | absolute baseline level (counts) | 700 | 700 |
| Alignment | *m/z* tolerance size | 0.1 | 0.1 |
|  | RT tolerance size (s) | 6 | 6 |
| Gap-filling | Intensity tolerance (%) | 20 | 20 |
|  | *m/z* tolerance size | 0.1 | 0.1 |
|  | RT tolerance size (s) | 6 | 6 |

**S2 Table. Method precisions (RSD) (*n*=6) of LC-MS analysis for serum and urine samples.**

|  | Compounds | Molecular Weight (Da) | Rt (min) | RSD (%) | Peak Area (×10^4^ cps) | RSD (%) |
| --- | --- | --- | --- | --- | --- | --- |
| **serum** | Adenosine | 267.24 | 1.37 ± 0.01 | 0.72 | 23.97 ± 1.71 | 7.13 |
|  | Tanshinone ⅡA | 294.33 | 21.82 ± 0.01 | 0.04 | 87.76 ± 3.66 | 4.17 |
|  | Tramadol | 263.37 | 6.95 ± 0.06 | 0.82 | 337.74 ± 8.28 | 2.45 |
|  | Isosorbide mononitrate | 236.14 | 21.98 ± 0.02 | 0.09 | 0.29 ± 0.01 | 4.05 |
|  | Digitoxin | 764.96 | 11.73 ± 0.01 | 0.10 | 0.31 ± 0.03 | 8.68 |
|  | Telmisartan | 514.62 | 12.96 ± 0.01 | 0.08 | 0.20 ± 0.03 | 14.30 |
| **urine** | Adenosine | 267.24 | 1.38 ± 0.01 | 0.37 | 26.70 ± 3.56 | 13.34 |
|  | Tanshinone ⅡA | 294.33 | 23.14 ± 0.01 | 0.02 | 70.86 ± 8.80 | 12.42 |
|  | Tramadol | 263.37 | 12.73 ± 0.04 | 0.31 | 226.52 ± 19.81 | 8.75 |
|  | Isosorbide mononitrate | 236.14 | 23.18 ± 0.01 | 0.05 | 0.13 ± 0.02 | 12.77 |
|  | Digitoxin | 764.96 | 21.18 ± 0.02 | 0.10 | 0.60± 0.07 | 11.78 |
|  | Telmisartan | 514.62 | 21.64 ± 0.01 | 0.06 | 0.41± 0.05 | 11.70 |

**S3 Table. Summary of potential biomarkers found between serum and urine samples of healthy rats and liver-injured rats by RRLC-ESIMS analysis in positive-ion mode.**

| **serum** | | | |  | **urine** | | | |
| --- | --- | --- | --- | --- | --- | --- | --- | --- |
| *m/z* | Rt(min) | trend*^a^* | *p* value |  | *m/z* | Rt(min) | trend*^a^* | *p* value |
| 118.0864 | 1.13 | **↓** | 2.07E-29 |  | 100.0739 | 4.96 | **↑** | 2.69E-04 |
| 118.0866 | 1.63 | **↑** | 2.02E-34 |  | 123.0441 | 2.76 | **↑** | 5.23E-04 |
| 123.0453 *^b^* | 2.74 | **↑** | 6.06E-07 |  | 123.0537 *^b^* | 1.86 | **↑** | 5.38E-04 |
| 130.0512 | 2.19 | **↑** | 2.33E-09 |  | 126.0867 | 1.98 | **↓** | 5.24E-12 |
| 132.0790 *^b^* | 1.22 | **↑** | 9.83E-11 |  | 130.0813 | 1.69 | **↓** | 9.68E-06 |
| 144.0815 *^b^* | 1.26 | **↓** | 3.44E-14 |  | 132.0765 *^b^* | 1.2 | **↑** | 9.56E-24 |
| 158.1178 | 1.71 | **↓** | 1.97E-10 |  | 138.0534 | 1.21 | **↓** | 3.43E-12 |
| 162.1129 | 1.16 | **↑** | 3.15E-13 |  | 140.0704 | 5.78 | **↓** | 1.26E-04 |
| 182.0837 *^b^* | 2.68 | **↑** | 1.77E-06 |  | 143.0794 | 1.26 | **↓** | 5.62E-05 |
| 188.0725 *^b^* | 5.2 | **↑** | 8.37E-07 |  | 143.1159 | 1.66 | **↓** | 1.74E-03 |
| 198.0861 | 1.14 | **↑** | 1.86E-20 |  | 144.0660 *^b^* | 4.02 | **↑** | 1.55E-05 |
| 204.1243 | 1.8 | **↓** | 1.28E-06 |  | 145.0999 | 1.29 | **↓** | 8.96E-04 |
| 205.1004 | 5.2 | **↑** | 1.21E-09 |  | 157.0933 | 3.82 | **↓** | 1.31E-05 |
| 220.0678 | 1.14 | **↑** | 4.93E-14 |  | 157.0957 | 2.4 | **↑** | 1.32E-04 |
| 355.2643 | 11.33 | **↑** | 1.72E-03 |  | 162.0532 | 11.1 | **↓** | 2.00E-09 |
| 466.3169 | 10.09 | **↑** | 7.64E-10 |  | 162.0766 | 1.25 | **↑** | 2.10E-16 |
| 482.3264 | 13.86 | **↓** | 4.69E-14 |  | 170.0592 | 14.34 | **↓** | 1.67E-08 |
| 482.3269 | 21.24 | **↑** | 1.12E-11 |  | 182.0803 *^b^* | 2.8 | **↑** | 9.94E-04 |
| 524.3579 | 21.39 | **↓** | 7.85E-09 |  | 184.0583 | 4.09 | **↓** | 4.19E-14 |
| 538.3873 | 22.21 | **↓** | 4.68E-17 |  | 185.1245 | 6.91 | **↓** | 7.13E-08 |
| 542.325 | 13.11 | **↓** | 4.26E-04 |  | 188.0692 *^b^* | 7.97 | **↑** | 1.71E-06 |
| 544.3317 | 14.53 | **↓** | 2.55E-10 |  | 189.1609 | 1.07 | **↑** | 2.58E-11 |
| 548.3717 | 18.68 | **↓** | 6.23E-06 |  | 190.0482 | 8.82 | **↓** | 2.86E-15 |
| 5,503,873 | 21.91 | **↓** | 2.29E-20 |  | 202.0451, | 9.8 | **↓** | 4.68E-06 |
| 560.336 | 11.42 | **↓** | 3.39E-12 |  | 206.0415 | 8.16 | **↓** | 9.00E-06 |
| 568.3405 | 14.48 | **↓** | 1.30E-17 |  | 212.1018 | 1.52 | **↓** | 1.17E-07 |
| 570.3552 | 15.43 | **↓** | 1.42E-10 |  | 215.0138 | 2.08 | **↓** | 2.20E-10 |
|  |  |  |  |  | 218.1128 | 2.34 | **↑** | 4.43E-18 |
|  |  |  |  |  | 220.1163 | 6.24 | **↑** | 2.47E-06 |
|  |  |  |  |  | 225.1077 | 11.76 | **↓** | 1.44E-08 |
|  |  |  |  |  | 226.0675 | 4.52 | **↑** | 4.15E-27 |
|  |  |  |  |  | 241.0919 | 5.05 | **↑** | 1.64E-19 |
|  |  |  |  |  | 245.159 | 3.39 | **↓** | 1.41E-09 |
|  |  |  |  |  | 245.16 | 2.97 | **↑** | 1.72E-23 |
|  |  |  |  |  | 254.1089 | 4.22 | **↑** | 1.70E-16 |
|  |  |  |  |  | 259.0897 | 1.51 | **↑** | 1.01E-15 |
|  |  |  |  |  | 265.0869 | 1.46 | **↑** | 1.70E-10 |
|  |  |  |  |  |  |  |  |  |
| **serum** |  | **urine** |  |  |  |  |  |  |
| *m/z* | Rt(min) | trend*^a^* | P value |  | *m/z* | Rt(min) | trend*^a^* | P value |
|  |  |  |  |  | 271.1372 | 2.87 | **↓** | 7.78E-08 |
|  |  |  |  |  | 312.1163 | 7.53 | **↑** | 3.69E-16 |
|  |  |  |  |  | 314.1208 | 1.82 | **↑** | 9.17E-07 |
|  |  |  |  |  | 333.1366 | 2.07 | **↑** | 3.99E-13 |
|  |  |  |  |  | 338.0837 | 9.96 | **↓** | 2.63E-05 |
|  |  |  |  |  | 343.1591 | 1.79 | **↑** | 2.28E-06 |
|  |  |  |  |  | 362.1163 | 2.04 | **↑** | 3.33E-03 |
|  |  |  |  |  | 376.1269 | 2.53 | **↑** | 1.54E-04 |
|  |  |  |  |  | 379.108 | 5.85 | **↑** | 5.05E-07 |
|  |  |  |  |  | 390.1129 | 1.71 | **↑** | 1.73E-03 |
|  |  |  |  |  | 404.0839 | 4.34 | **↑** | 9.95E-20 |
|  |  |  |  |  | 418.137 | 7 | **↑** | 3.70E-05 |
|  |  |  |  |  | 74.0590, | 1.12 | **↑** | 1.34E-04 |
|  |  |  |  |  | 74.0593 | 1.4 | **↑** | 1.86E-04 |
|  |  |  |  |  | 86.0584 | 1.95 | **↓** | 5.53E-07 |
|  |  |  |  |  | 86.0596 | 2.88 | **↑** | 8.62E-24 |

*^a^* The concentration trend of potential biomarkers found between healthy control and liver injury group, ↓ indicates a relatively higher concentration present in healthy controls and a relatively lower concentration in the liver-injured group.

↑ indicates a relatively lower concentration present in healthy controls and an increased concentration in the liver-injured group.

*^b^* Identified in both serum and urine.

**Supplementary materials**

[Self-compiling program](http://dict.cnki.net/dict_result.aspx?searchword=%e8%87%aa%e7%bc%96%e7%a8%8b%e5%ba%8f&tjType=sentence&style=&t=self-compiling+program) derived from Microsoft Visual basic

Sub main()

Dim r As Long, a As Long, b As Long

Dim MZ As Double, RT As Double

With Application

.Calculation = xlCalculationManual

.ScreenUpdating = False

MZ = Abs(Application.InputBox(prompt:="M/Z TOLERANCE(value)：", Title:="M/Z", Type:=1))

RT = Abs(Application.InputBox(prompt:="RT TOLERANCE (Value)：", Title:="RT", Type:=1))

r = ActiveSheet.UsedRange.Rows.Count

Range(Cells(3, 7), Cells(r, 10)).ClearContents

For a = 3 To r

For b = 3 To r

If Abs(Cells(a, 1) - Cells(b, 3)) <= MZ And Abs(Cells(a, 2) - Cells(b, 4)) <= RT Then

Cells(a, 7) = Cells(a, 1)

Cells(a, 8) = Cells(a, 2)

Cells(a, 9) = Cells(b, 3)

Cells(a, 10) = Cells(b, 4)

Cells(a, 1).Interior.ColorIndex = 3

Cells(a, 2).Interior.ColorIndex = 3

Cells(b, 3).Interior.ColorIndex = 5

Cells(b, 4).Interior.ColorIndex = 5

Exit For

ElseIf Abs(Cells(a, 1) - Cells(b, 3)) > MZ Or Abs(Cells(a, 2) - Cells(b, 4)) > RT Then

End If

Next b

Next a

End With

End

**S1 Fig. Overlaid extracted ion chromatograms of standard compounds in various test mixtures using RRLC–MS analysis during the whole sample batch (n = 6). Six standard compounds, (a) Adenosine, (b) Tanshinone IIA, (c) Tramadol, (d) Isosorbide mononitrate, (e) Digitoxin, and (f) Telmisartan, were selected to prepare various test mixtures for the (A) serum and (B) urine samples. They all provide an overall idea of the system stability during the run.**


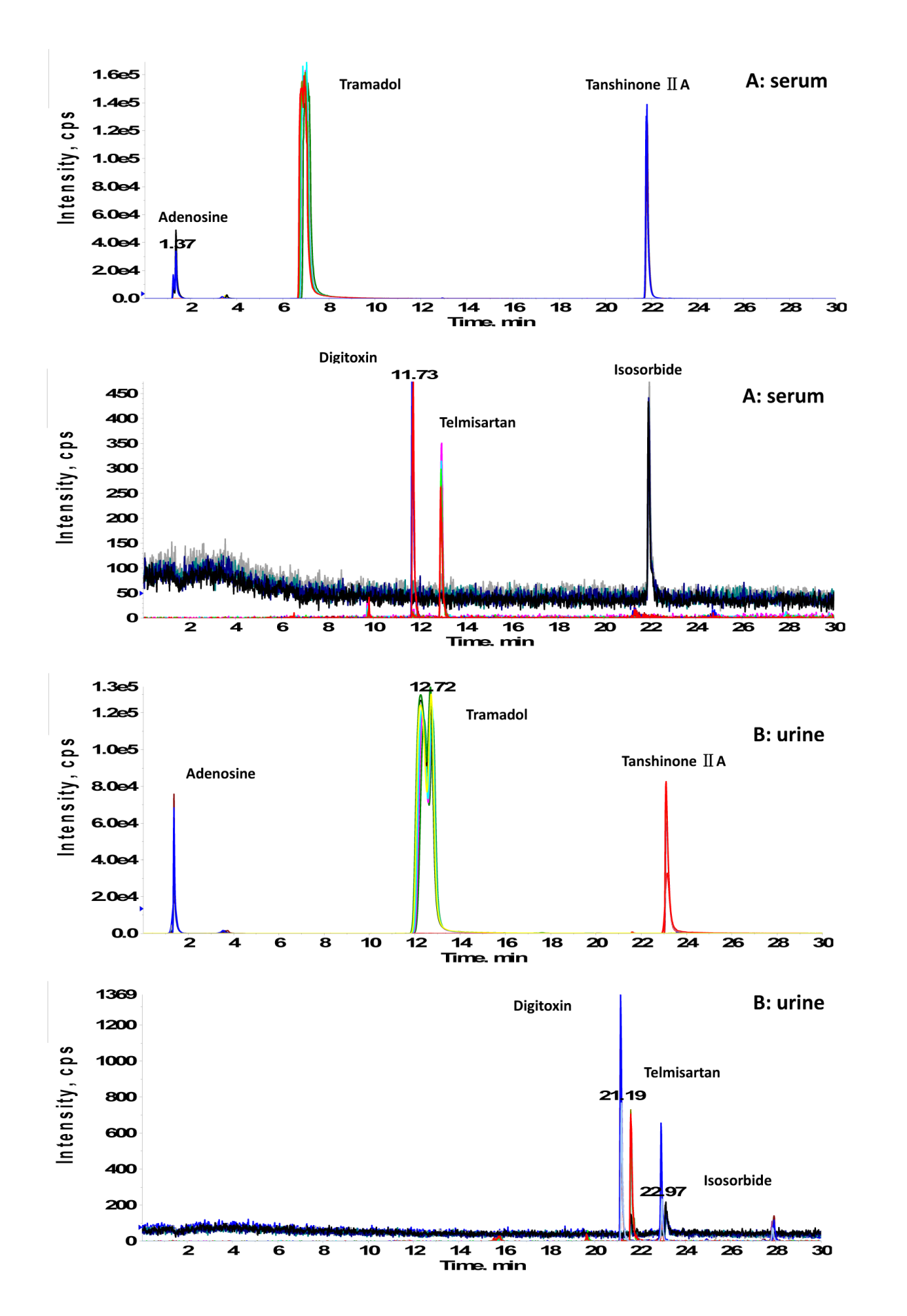


**S2 Quality control (QC) plots of six randomly repeated runs of RRLC–(+) ESIMS generated by principle component analysis for serum (A) and urine (B). Peak area deviation could be evaluated by distribution of the runs. X-axis: run order; Y-axis: standard deviation. (A1) and (B1) are QC plot of principle component analysis; (A2) and (B2) are QC plot for the first component. (A3) and (B3) are QC plot for the second component.**


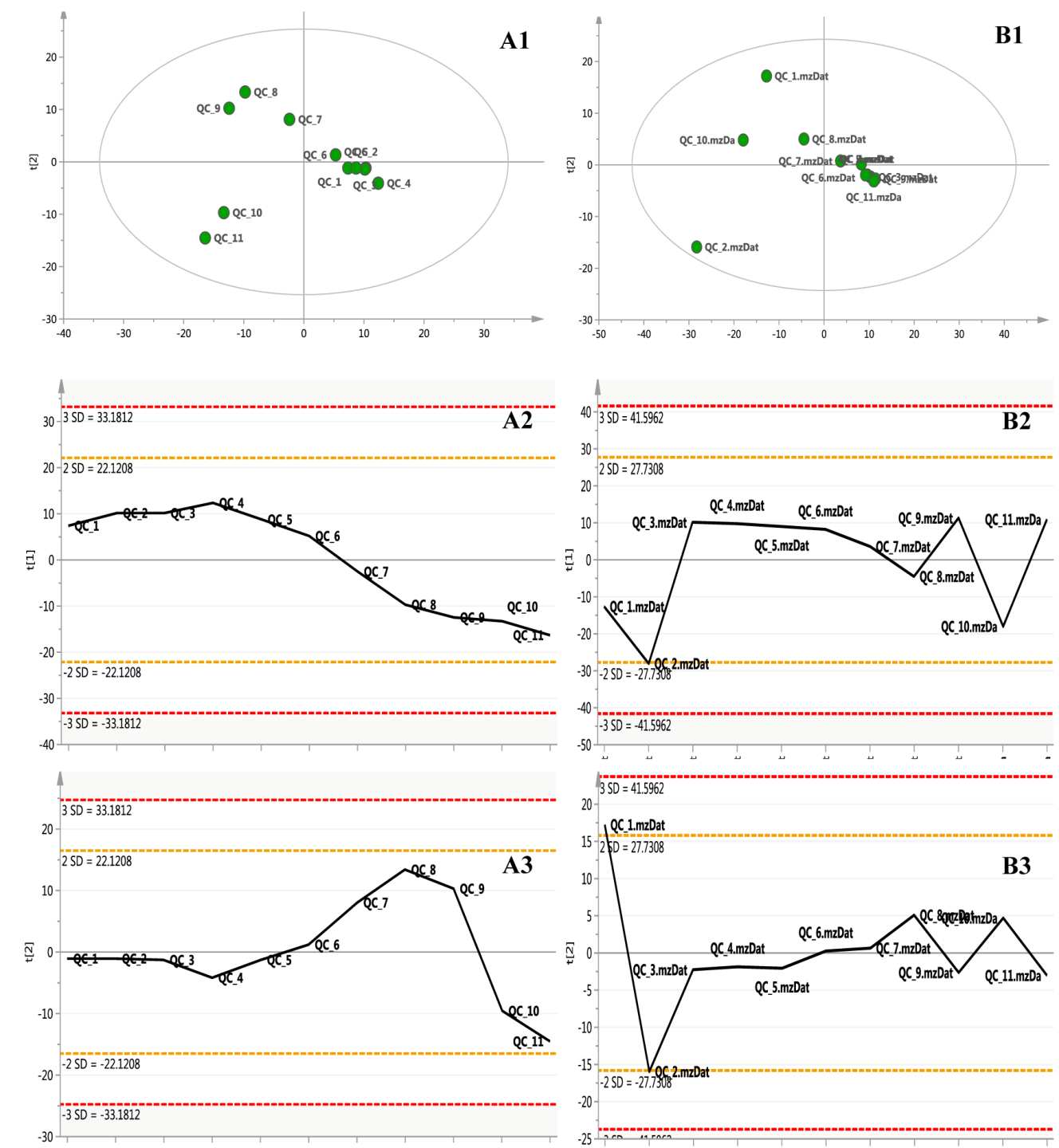


**S3 Fig. Validation plots of the PLS-DA models obtained by using 100 permutation tests and T-predicted scatter plots of the OPLS-DA model for serum (A–B) and urine (C–D) samples. (Symbols indicate ● = C healthy rats; ● = M liver-injured rats induced by hydrazine at 24 h post-dosing and ● = FM liver-injured rats induced by hydrazine at 48 h post-dosing).**

**S4 Fig. Visualization of the discriminatory powers of potential biomarkers. (A) The seven upregulated metabolites provided AUC values of 0.667 to 1. (B) The** **eleven downregulated metabolites provided AUC values of 0.838 to 1.**


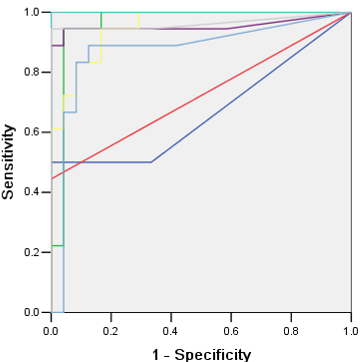

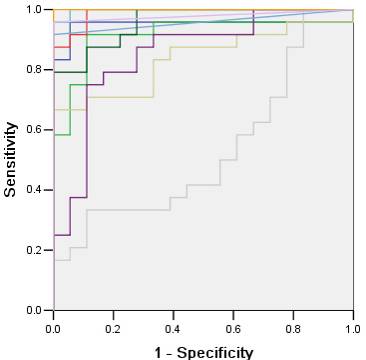


**A**

**B**

**S5 Fig. ROC curve of the combined potential biomarkers (AUC>0.85) which provided an AUC of 1 and sensitivities and specificities reached 100% respectively.**


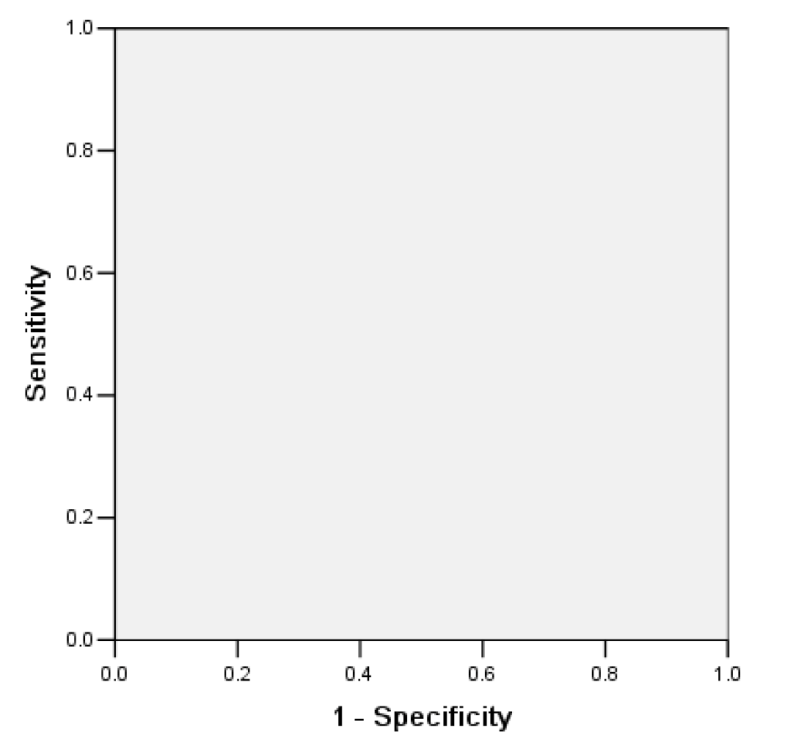

Supplement: Supplementary Materials — S1 Table: the parameters of detailed preprocessing data applied in MZmine. S2 Table: method precisions (RSD) (n = 6) of LC-MS analysis for serum and urine samples. S3 Table: summary of potential biomarkers found between serum and urine samples of healthy rats and liver-injured rats by RRLC-ESIMS analysis in positive-ion mode. S1 Fig: overlaid extracted ion chromatograms of standard compounds in various test mixtures using RRLC-MS analysis during the whole sample batch (n = 6). Six standard compounds, (a) adenosine, (b) tanshinone IIA, (c) tramadol, (d) isosorbide mononitrate, (e) digitoxin, and (f) telmisartan, were selected to prepare various test mixtures for the (A) serum and (B) urine samples. They all provide an overall idea of the system stability during the run. S2 Fig: quality control (QC) plots of six randomly repeated runs of RRLC–(+) ESIMS generated by principal component analysis for serum (A) and urine (B). Peak area deviation could be evaluated by the distribution of the runs. x-axis: run order; y-axis: standard deviation. (A1) and (B1) are QC plot of principal component analysis; (A2) and (B2) are QC plot for the first component. (A3) and (B3) are QC plot for the second component. S3 Fig: validation plots of the PLS-DA models obtained by using 100 permutation tests and T-predicted scatter plots of the OPLS-DA model for serum (A-B) and urine (C-D) samples. (The symbols are as follows: red circle = C healthy rats; brown circle = M liver-injured rats induced by hydrazine at 24 h postdosing; and blue circle = FM liver-injured rats induced by hydrazine at 48 h postdosing.) S4 Fig: visualization of the discriminatory powers of potential biomarkers. (A) The eight upregulated metabolites provided AUC values of 0.667 to 1. (B) The 11 downregulated metabolites provided AUC values of 0.838 to 1. S5 Fig: ROC curve of the combined potential biomarkers (AUC > 0.85) that provided an AUC of 1 and sensitivities and specificities that reached 100%, respectively. [file 8473161.f1.docx]
